# Large enhancement of red upconversion luminescence in beta Ba_2_Sc_0.67_Yb_0.3_Er_0.03_AlO_5_ phosphor via Mn^2+^ ions doping for thermometry

**DOI:** 10.1038/s41598-024-59732-x

**Published:** 2024-04-17

**Authors:** Yongtao Liu, Bin Duan, Lin Zhou, Yuxiang Wu, Fengyi Wang, Changchun Ding, Junshan Hu

**Affiliations:** 1 School of Science, Xihua University, Chengdu, 610039 China; 2 College of Materials Science and Engineering, Sichuan University, Chengdu, 610065 China

**Keywords:** Upconversion luminescence, Mn^2+^ doping, Single red emission, Optical thermometry, Nonlinear optics, Nonlinear optics

## Abstract

Here, this study reports single-band red upconversion emission in β-Ba_2_ScAlO_5_: Yb^3+^/Er^3+^ phosphor by doping Mn^2+^. The optimum concentration of Mn^2+^ ions in β-Ba_2_ScAlO_5_: Yb^3+^/Er^3+^ phosphor was 0.20. The intensity of red and green emissions is increased by 27.4 and 19.3 times, respectively. Compared with the samples without Mn^2+^ ions, the red-green integral strength ratio of β-Ba_2_ScAlO_5_: Yb^3+^/Er^3+^/Mn^2+^ sample was significantly increased by 28.4 times, reaching 110.9. The UCL mechanism was explored by analyzing the down-conversion luminescence spectra, absorption spectra, UCL spectra, and upconversion fluorescence lifetime decay curves of Yb^3+^/Er^3+^/Mn^2+^ co-doped β-Ba_2_ScAlO_5_. The enhancement of upconversion red light is achieved through energy transfer between defect bands and Er^3+^ ions, as well as energy transfer between Mn^2+^ ions and Er^3+^ ions. In addition, the Mn^2+^ doped β-Ba_2_ScAlO_5_: Yb^3+^/Er^3+^ red UCL phosphors have great potential for ambient temperature sensing in the 298–523 K temperature range. The maximum sensitivity of β-Ba_2_ScAlO_5_: Yb^3+^/Er^3+^/Mn^2+^ phosphor as a temperature sensor at 523 K is 0.0247 K^−1^.

## Introduction

In recent years, rare-earth-doped upconversion luminescence (UCL) materials have been widely used in optical anticounterfeiting, optical temperature measurement, and illumination display due to their stable physicochemical properties, low toxicity, and long fluorescence lifetime^1–6^. At present, the UCL efficiency is still one of the focuses of research, but the single-color UCL has gradually become one of the focuses of research at this stage, especially to achieve the single UCL of red and near-infrared light^7,8^. In the UCL process, rare-earth Yb^3+^ ions are widely used as sensitizers due to their strong absorption cross section near 980 nm. Meanwhile, Er^3+^ ions have abundant step energy states that can be coupled with sensitizers and become commonly used as activators^9,10^. Upconversion materials co-doped with Yb^3+^ and Er^3+^ ions tend to emit strong green light and relatively weak red light^11^. Unfortunately, the poor penetration of green luminescence into biological tissues limits the biomedical applications of this class of materials, whereas light located in the red to near-infrared wavelength band (650–1350 nm) has a strong biological tissue penetration capability^12,13^. Therefore, red UCL materials are very interesting for optical temperature measurement, bio-imaging, and medical diagnostics^14–16^. Optical temperature measurement technology, due to its non-contact measurement, fast response and high sensitivity, has shown great application potential in daily life, medical treatment, scientific research and other fields^17,18^. Optical temperature sensing is a technology to obtain temperature information by measuring the change of optical properties of objects, including fluorescence intensity, fluorescence ratio and decay lifetime^19,20^. The single emission temperature measurement is based on the change of the relationship between a certain luminous intensity and temperature in the luminous center of the material. The application of upconversion green emission luminescence technology in optical temperature measurement has been studied, but the penetration of green light in complex environments and biological tissues is poor^21,22^. Therefore, it is urgent to develop upconversion red light emission materials for optical temperature measurement.

Various techniques have been employed to modulate the UCL performance and single-colour UCL, such as substrate selection, rare-earth ion doping type, concentration, crystal field modulation, surface plasmon, and doping of transition metals^23–27^. Wang et al*.* synthesized a novel K_3_(Y_0.88_Yb_0.10_Er_0.02_)Si_2_O_7_ phosphor, which enhanced the UCL intensity and thus increased the absolute and relative sensitivity^28^. Lin and colleagues designed a novel spindle probe with an adjustable aspect ratio for mitochondrial imaging and coated gold nanoparticles (SPS@Au) layer by layer on the surface of the probe, which has the property of enhancing the red UCL and can be used for synergistic immune-photodynamic anticancer therapy^29^. Bai et al*.* gave the CeO_2_: Yb/Er samples stronger UCL by doping transition metal Fe^3+^ ions, which enhanced the photoelectric conversion efficiency^30^. In addition, Bi et al*.* achieved single-band red light emission and improved the red to green ratio (R/G) by adjusting the Mn^2+^ content in NaLnF_4_: Er/Mn^31^. Recently, we found that a new β-Ba_2_ScAlO_5_: 0.3Yb^3+^/0.03Er^3+^ (BASO: Y/E) phosphor could achieve red upconversion emission^32^. However, the upconversion red emission intensity of BASO: Y/E phosphors is still low and cannot satisfy the optical temperature measurement. Therefore, we need to further increase the intensity of red light emission to meet current needs.

This study achieved an intense single-band red UCL by doping Mn^2+^ in BASO: Y/E samples. The study results show that the red emission was enhanced in the optimum sample of 0.20 Mn^2+^, and the red to green integral intensity ratio reaches a high value of 110.9. The UCL mechanism was explored by analyzing the down-conversion luminescence spectra, absorption spectra, UCL spectra, and upconversion fluorescence lifetime decay curves of β-Ba_2_ScAlO_5_: Yb^3+^/Er^3+^/Mn^2+^ (BSAO: Y/E/M) phosphors. The enhancement of upconversion red light is achieved through energy transfer between defect bands and Er^3+^ ions, as well as energy transfer between Mn^2+^ ions and Er^3+^ ions. The BSAO: Y/E/M phosphors have a wide range of applications in optical temperature measurement.

## Experimental

### Materials

BSAO: Y/E/M phosphors were synthesized using a solid-phase method at high temperatures^32^. Primary raw materials, including high-purity BaCO_3_, Sc_2_O_3_, Al(OH)_3_, Mn_2_O_3_, Yb_2_O_3_, and Er_2_O_3_, were directly purchased from Aladdin Corporation without additional refinement.

### Synthesis

Unprocessed materials (BaCO_3_, Sc_2_O_3_, Al(OH)_3_, Mn_2_O_3_, Yb_2_O_3_, and Er_2_O_3_) were weighed in a beaker according to stoichiometry. The mixture was then placed in a blender with anhydrous ethanol and stirred for 1 h to create a well-mixed suspension. After standing at ambient temperature for 2 h and incubating at 70 °C for 24 h, the dried raw materials were ground in an agate bowl and pressed into tablets. The samples were subjected to a high-temperature reaction furnace: temperature was raised to 900 °C for 30 min, then to 1700 °C for 1.5 h^33,34^. Finally, the temperature was lowered to ambient temperature. The samples were ground in an agate bowl to obtain BSAO: Y/E/M phosphors.

### Instruments

X-ray diffractograms of prepared compounds were obtained using a diffractometer (BRUKER D8 ADVANCED, MA, Germany, Cu target Kα radiation, *λ* = 1.54184 Å) in the 2θ range of 8° to 75° at steps of 0.02°. Shape and size of prepared materials were examined using a Japan JEOL JSM6701F field emission scanning electron microscope (FESEM). Elemental composition was analyzed using a JEOL JED-2300 energy dispersive spectrometer (EDS) attached to a FESEM. A fluorescence spectrophotometer (Acton Spectra Pro 2300i) with a 980 nm near-infrared laser emitter was used to test the 300–800 nm spectral range by Princeton Instruments, USA. The absorption spectrum was characterized using the Lambda 750 absorption spectrometer by Perkin Elmer, USA^35,36^.

## Results and discussion

**Figure 1:**
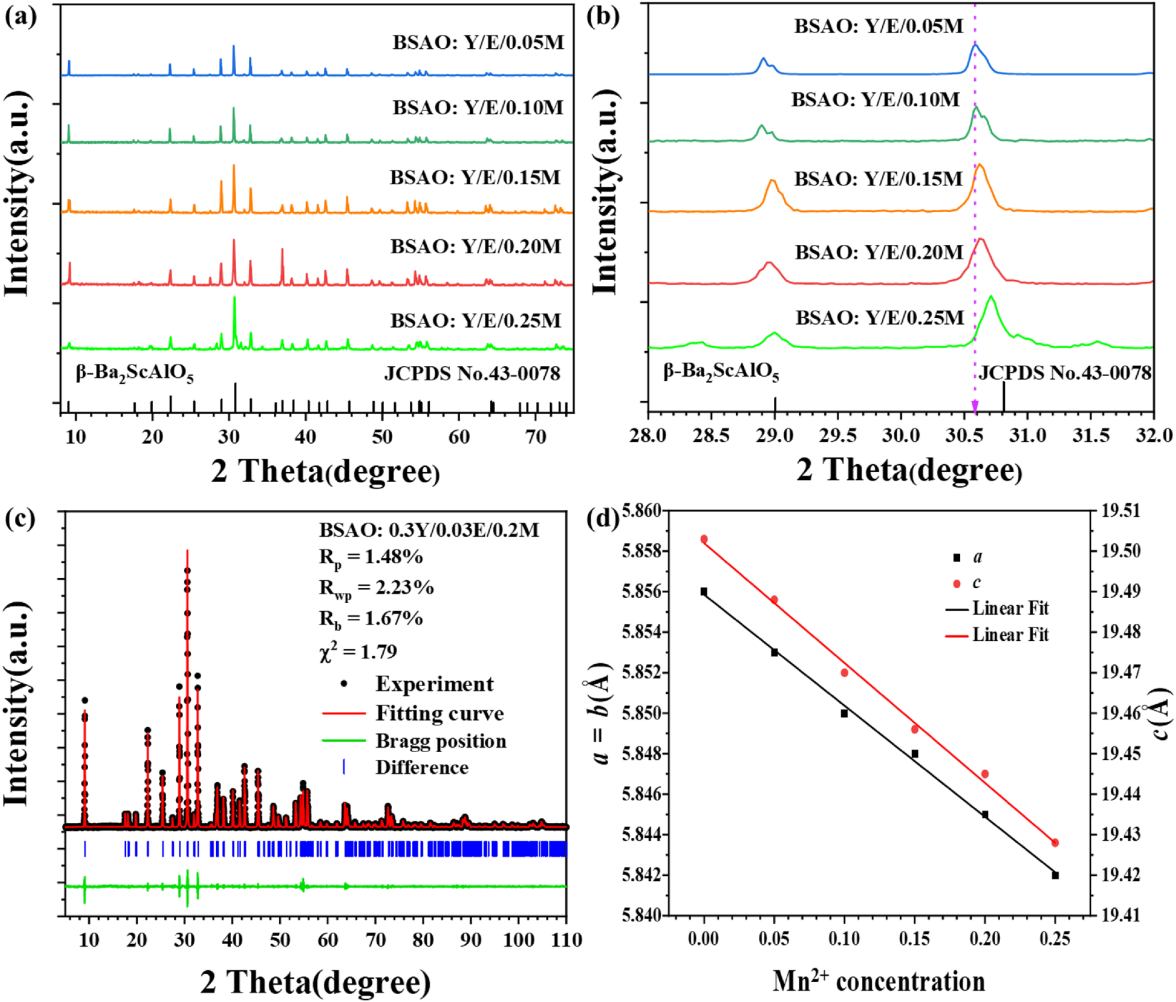
(**a**,**b**) XRD patterns and partial enlargement of different concentrations of BSAO: Y/E/M, (**c**) the refined XRD patterns of BSAO: Y/E/0.2 M and (**d**) cell parameters of different concentrations of BSAO: Y/E/M.

Figure 1a shows the XRD diffraction spectra of BASO: Y/E phosphor doped with different concentrations of *x*Mn^2+^ (*x* = 0.05, 0.10, 0.15, 0.20 and 0.25) and β-Ba_2_ScAlO_5_ standard card (JCPDS No. 43-0078). The diffraction peaks of all samples agree with the diffraction pattern of pure phase β-Ba_2_ScAlO_5_ of JCPDS No. 43–0078 [space group *P*6_3_/mmc (194)]^37^. This result confirms the effective doping of Mn^2+^, Yb^3+^ and Er^3+^ ions in β-Ba_2_ScAlO_5_ phosphor. All the diffraction peaks of the XRD of the samples were shifted to a high angle with increasing Mn^2+^ ion doping concentration (see Fig. 1b). Rietica program was used to perform Rietveld refinement calculations on cell parameters of BSAO: Y/E/M, and the results are shown in Fig. 1c,d. The unit parameters and atomic coordinates after refinement are shown in Table 1. The results show that the ionic radius of Mn^2+^ (0.67 Å, CN = 4) is smaller than that of Ba^2+^ (1.34 Å, CN = 4), and the parameters *a* and *c* decrease with the increase of Mn^2+^ content. The refinement of atomic coordinates and position occupation reveal the changes of internal structure of Mn^2+^ doped crystals: (1) Mn^2+^ ions occupy Ba^2+^ 2a, 4f. and Al^3+^ 4e sites simultaneously; (2) The oxygen vacancy content of the doped Mn^2+^ sample was 6.2%, which was significantly higher than that of the undoped Mn^2+^ sample (1.3%). Structural changes usually result in the optical properties of the material^33,34^.

**Table 1:** Refined results of the (Ba_1−*x*_Mn_*x*_)_2_(Sc_0.67_Yb_0.3_Er_0.03_)AlO_5_ samples with *x* = 0 and 0.2. The concentration of rare earth Er^3+^ in the compounds is so low (3 at% of the Sc^3+^ site) that it is not included in the refinement.

Parameter	Sample								
	*x* = 0	*x* = 0.2							
Space group	*P* 6_3_/mmc								
Cell constant (Å)	*a* = *b* = 5.856, *c* = 19.503	*a* = *b* = 5.845, *c* = 19.445							
Overall thermal displacement (Å^2^)	0.793	1.113							

Atom	Site	*x*	*y*	*z*	*n*	*x*	*y*	*z*	*n*
Sc	4f	0.33333	0.66667	0.56476	0.11891	0.33333	0.66667	0.56371	0.11543
Ba	2a	0.00000	0.00000	0.00000	0.08333	0.00000	0.00000	0.00000	0.06101
Ba	2d	0.33333	0.66667	0.75000	0.07741	0.33333	0.66667	0.75000	0.08333
Ba	4f	0.33333	0.66667	0.38956	0.16667	0.33333	0.66667	0.38951	0.15266
Al	4e	0.00000	0.00000	0.16417	0.16667	0.00000	0.00000	0.17139	0.15002
O	12k	0.16600	0.33200	0.63406	0.50000	0.16600	0.32755	0.62816	0.42051
O	2b	0.00000	0.00000	0.25000	0.05938	0.00000	0.00000	0.25000	0.08333
O	6 g	0.50000	0.00000	0.00000	0.21517	0.50000	0.00000	0.00000	0.20332
Yb	4f	0.33333	0.66667	0.56476	0.04776	0.33333	0.66667	0.56371	0.05124
Mn	2a	–	–	–	–	0.00000	0.00000	0.00000	0.02232
Mn	4f	–	–	–	–	0.33333	0.66667	0.38951	0.01401
Mn	4e	–	–	–	–	0.00000	0.00000	0.17139	0.01665
Oxygen vacancy	~ 1.3%	~ 6.2%							

**Figure 2:**
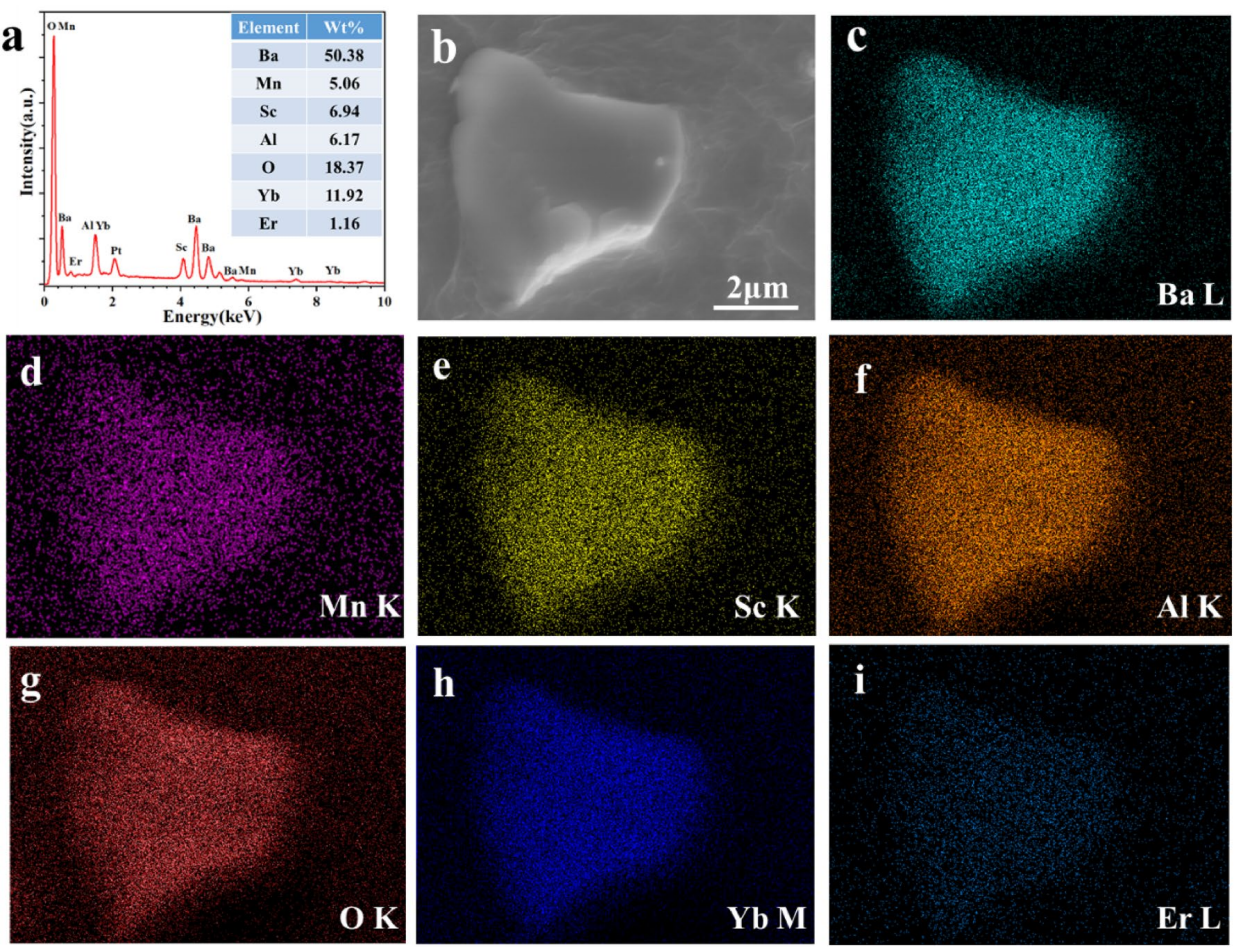
(**a**) and (**b**) show the EDS and FESEM images of BSAO: Y/E/0.2 M phosphor, respectively. The EDS mapping depicted in (**c**–**i**) provides a detailed profile of the chemical composition of BSAO: Y/E/0.2 M phosphor.

The morphology and elemental composition of the BSAO: Y/E/0.2 M sample were characterized using FESEM and EDS. The mass fractions of Ba, Mn, Sc, Al, O, Yb and Er elements are very close to the theoretical values of 58.38%, 5.06%, 6.94%, 6.17%, 18.37%, 11.92%, and 1.16%, respectively, in Fig. 2a. The EDS spectra show that they are uniformly distributed in the phosphor in Fig. 2b–i. The results show that Yb^3+^, Er^3+^ and Mn^2+^ ions were successfully doped into the β-Ba_2_ScAlO_5_ lattice, reducing concentration quenching and improving UCL efficiency.

**Figure 3:**
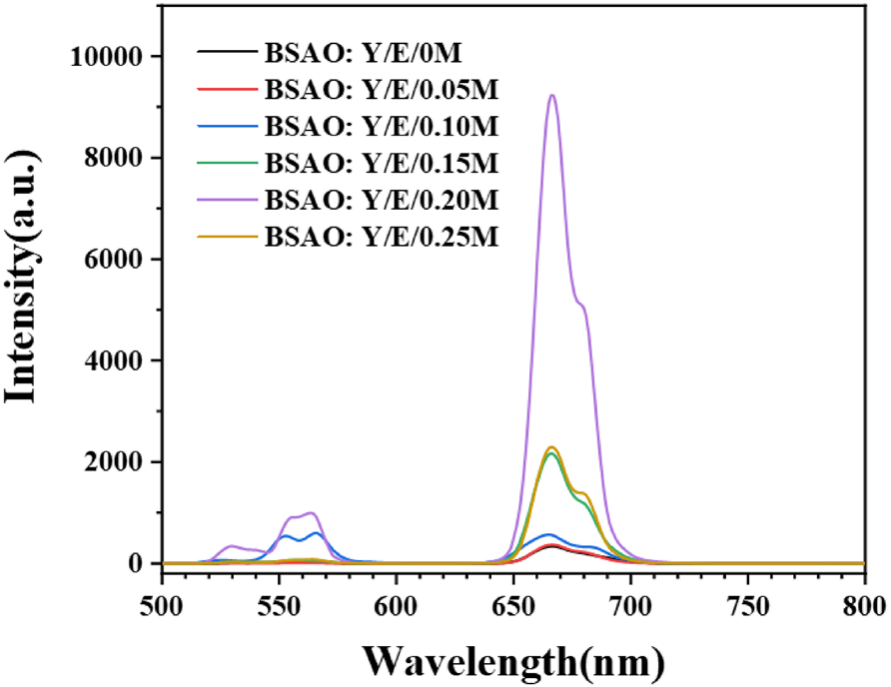
The UCL spectra of BSAO: Y/E/*x*M (*x* = 0–0.25) excited by a 980 nm laser with a power of 300 mW.

Figure 3 shows the UCL spectra of the BSAO: Y/E/*x*M (*x* = 0–0.25) phosphor under 980 nm laser excitation. Weak green and strong red emission peaks appeared at 530 nm, 566 nm and 664 nm, respectively. The green (530 nm and 566 nm) and red (664 nm) upconversion emission of BSAO: Y/E/M samples corresponds to the radiative transitions of the Er^3+^ energy levels ^2^H_11/2_ → ^4^I_15/2_, ^4^S_3/2_ → ^4^I_15/2_ and ^4^F_9/2_ → ^4^I_15/2_, respectively. The increase of Mn^2+^ concentration enhances the upconversion red emission intensity of the sample. With the increase of Mn^2+^ concentration, the luminous intensity of the upconversion red emission was first enhanced and then weakened. When the Mn^2+^ concentration was 0.20, the red emission reached the strongest. The intensity of red and green emissions is increased by 27.4 and 19.3 times, respectively. At a doping concentration of 0.20, the ratio reaches 110.9, an increase of 28.4 compared to the undoped Mn^2+^ sample, which has a ratio of 82.5^32^. A single red upconversion emission was achieved for BSAO: Y/E/0.20 M.

**Figure 4:**
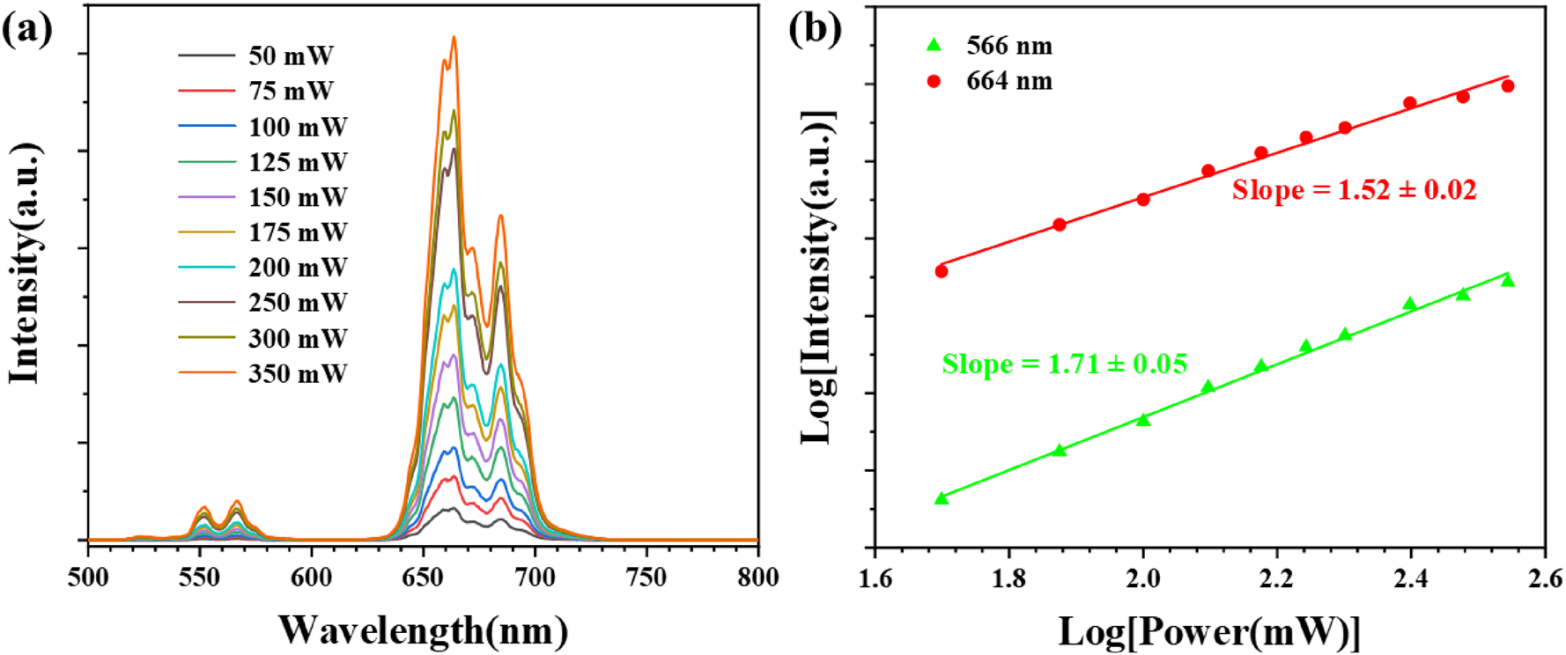
(**a**) The UCL spectrum of BSAO: Y/E/0.2 M under 980 nm laser excitation versus pump power; (**b**) Pump power dependence of the green and red luminescence intensity of BSAO: Y/E/0.2 M.

In order to explore the luminescence mechanism of BSAO: Y/E/*x*M (*x* = 0–0.25) phosphor, the power dependence of BSAO: Y/E/0.2 M under 980 nm laser excitation was investigated. In Fig. 4a, the upconversion red-green luminescence intensity of BSAO: Y/E/0.2 M phosphor is enhanced with increased excitation power. Meanwhile, the mathematical relationship between the UCL intensity (*I*) and the pump power (*P*) is given by^38^: *I* ∝ *P*^*n*^. The exponent *n* denotes the number of photons required for the upconversion process. The double logarithmic coordinate plots from the above equation are shown in Fig. 4b, and the slopes of the fitted curves indicate that the quantum numbers *n* required for UCL at 664 nm and 566 nm are 1.52 and 1.71, respectively. The *n* values are close to 2, which suggests that the ^4^S_3/2_ and ^4^F_9/2_ energy levels of the Er^3+^ ion are involved in the two-photon process. The numerical coefficients *n* associated with the red and green UCL of BSAO: Y/E are 1.46 and 1.57, respectively^32^. Doping of the Mn^2+^ ion increases the number of photons emitted by red light from 1.46 to 1.52 and that emitted by green light from 1.57 to 1.71. Interestingly, although the number of photons increases in both cases, the number of photons for the green light is significantly larger than that for the red light, suggesting that the doping of Mn^2+^ ions may have changed the UCL mechanism^39,40^.

**Figure 5:**
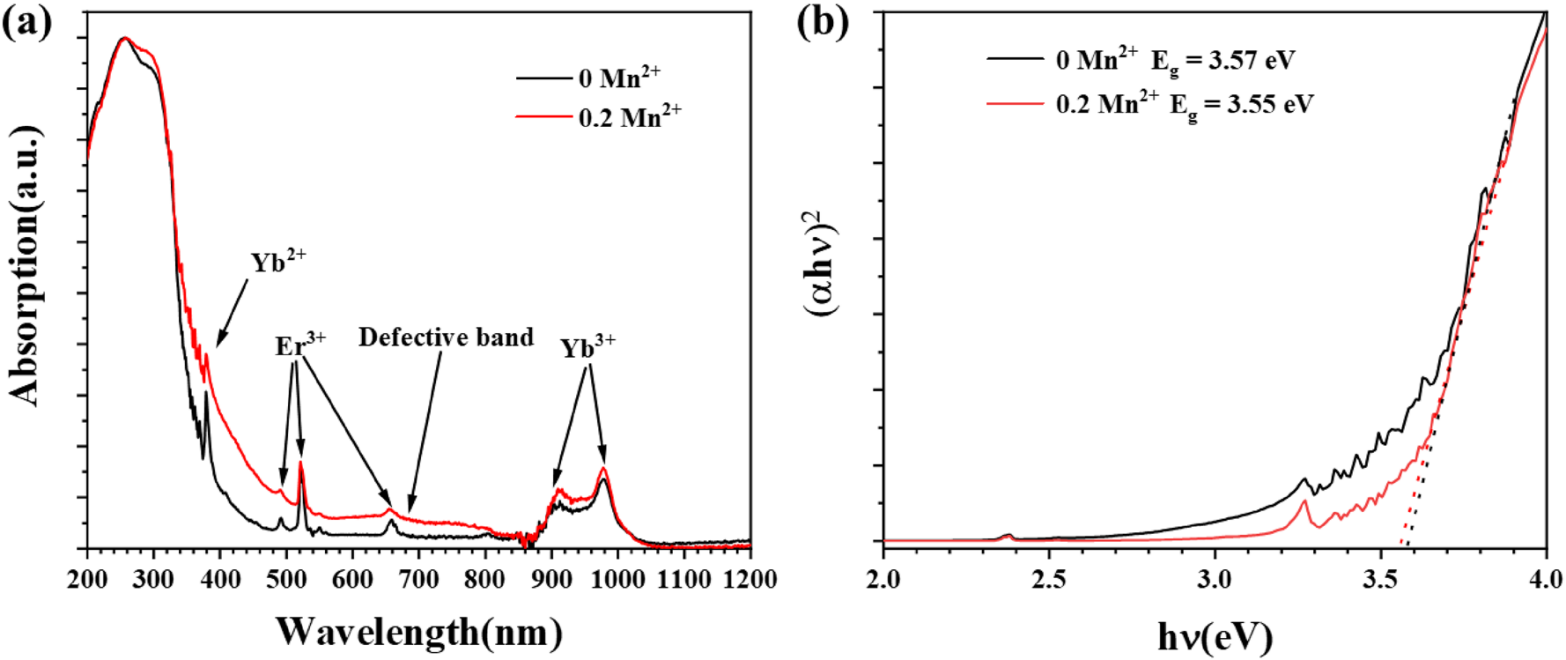
(**a**) Absorption spectra and (**b**) graph of (*αhυ*)^2^ versus *hυ* correspondence of BSAO: Y/E/*x*M (*x* = 0, 0.2).

In order to investigate the upconversion red luminescence enhancement mechanism of BSAO: Y/E/M phosphor, its absorption spectra were characterized as shown in Fig. 5. Figure 5a shows the absorption spectra of BSAO: Y/E/*x*M (*x* = 0, 0.2). The two samples have six identical absorption bands at the same location: one broad band (main peak at 300 nm) and five peaks (380, 488, 530, 664, and 980 nm). The absorption band near 300 nm is the intrinsic absorption of the Ba_2_ScAlO_5_ main matrix. The absorption peaks at 488, 530 and 664 nm are the characteristic absorption of the rare-earth Er^3+^ ions. The absorption peak at 980 nm is from the rare-earth Yb^3+^ ion. The absorption peak at 380 nm is from the Yb^2+^ ion. The change in the valence state of the Yb ion from + 3 to + 2 results in a decrease in the number of positive charges in the crystal^41^. Therefore, the increase in oxygen vacancies is necessary to maintain the electroneutrality of the material^42^. Furthermore, a broad absorption band from 470 to 820 nm is observed in BSAO: Y/E/M doped with 0.2 Mn^2+^ ions compared to the undoped Mn^2+^ sample. The center wavelength is about 640 nm. The broad absorption band ranging from 470 to 820 nm is due to oxygen vacancy defects caused by doping with 0.2 Mn^2+^. Figure 5b shows the correspondence between (*αhυ*)^2^ and *hυ* for BSAO: Y/E/*x*M (*x* = 0, 0.2) samples. The calculated results show that the band gap of Mn^2+^ doped is 3.55 eV, and that of undoped Mn^2+^ is 3.57 eV^43–45^. Therefore, the band gap can be reduced by doping Mn^2+^ ions, which confirms the above conclusion.

**Figure 6:**
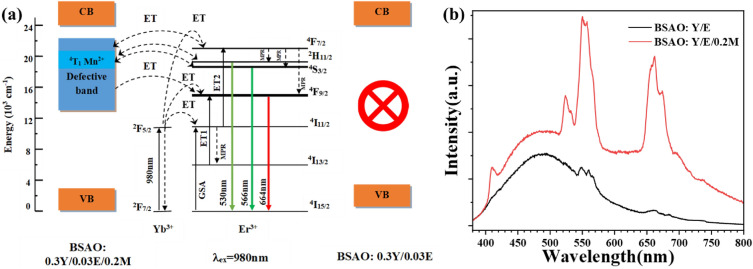
(**a**) The energy leap mechanism of BSAO: Y/E/0.2 M. (**b**) The photoluminescence spectra of BSAO: Y/E/0.2 M under 365 nm laser excitation.

The upconversion excitation processes mainly include multiphonon relaxation (MPR), ground state absorption (GSA), and energy transfer (ET) processes^46^. Figure 6a shows the conversion mechanism of BSAO: Y/E/M phosphor, demonstrating its energy transfer paths. Based on a previous report, we demonstrated that the main excitation pathway for the red upconversion emission is GSA + MPR + ET1^32^. In the BSAO: Y/E/0.2 M phosphor, the broad absorption band from 470 to 820 nm is due to oxygen vacancy defects. As shown in Fig. 6a, the defect bands coincide precisely with the emission levels in red (^4^F_9/2_) and green (^2^H_11/2_ and ^4^S_3/2_), which leads to the energy transfer process between the defect bands and the corresponding energy levels of Er^3+^ ions (^4^F_7/2_, ^4^F_9/2_, ^2^H_11/2_ and ^4^S_3/2_). The first energy transfer pathway for enhanced UCL: the energy of the ^4^F_7/2_ energy level of Er^3+^ is transferred to the defect band through energy transfer, and then the defect band is transferred to the ^2^H_11/2_, ^4^S_3/2_, and ^4^F_9/2_ energy levels through energy transfer to emit the corresponding green (530 and 566 nm) and red (664 nm) light. Since the ^4^F_9/2_ energy level is located at the edge of the absorption band, the defect band transfers more energy to the ^4^F_9/2_ energy level^47^. This finding is consistent with the significant enhancement of red light in the UCL spectrum (Fig. 3). The second energy transfer pathway for enhanced UCL: the non-radiative energy transfer of Er^3+^ to Mn^2+^ (^2^H _11/2_/^4^S_3/2_ → ^4^T_1_), followed by the back energy transfer to Er^3+^ (^4^T_1_ → ^4^F_9/2_)^48^. As the content of Mn^2+^ increases, the weak green radiation of Er^3+^ hardly changes, while the red radiation is greatly enhanced, indicating that the energy transfer between Er^3+^ and Mn^2+^ is very efficient. As a result, higher R/G ratios can be achieved.

Figure 6b shows the down-conversion luminescence spectra of BSAO: Y/E and BSAO: Y/E/0.2 M. A broad emission band centered at 485 nm is observed for both samples beside the characteristic emission peaks of rare earth Yb^3+^ and Er^3+^. This emission band corresponds to the conduction band to the valence band leap of the two samples. As can be seen from the black line in Fig. 6b, the BSAO: Y/E phosphor exhibits a strong green emission under 365 nm excitation with a green–red ratio of 2.97. After Mn^2+^ doping, BSAO: Y/E/0.2 M has a green emission peak and a red emission peak [red line in Fig. 6b] with a green–red ratio of 0.95. Under 365 nm laser excitation, BSAO: Y/E/0.2 M directly leaps the energy of Er^3+^ in the ^4^F_7/2_ energy level to the ^2^H_11/2_, ^4^S_3/2_, and ^4^F_9/2_ excited states via MPR. The emission of red light indicates that more photons relax to the ^4^F_9/2_ excited state, while very few photons go to the ^2^H_11/2_ and ^4^S_3/2_ energy levels, so the red light is stronger and the green light is weaker. The BSAO: Y/E shows red light under 980 nm excitation with a red-green ratio of about 82.5^32^. For the BSAO: Y/E/0.2 M sample, red emissions were enhanced under 980 nm laser excitation, and a high R/G ratio of 110.9 was observed. This shows that the upconversion emission excitation path of BSAO: Y/E/0.2 M does not change compared with BSAO: Y/E, only two energy transfer paths are added to increase the number of electrons at the luminous level, thus enhancing the upconversion emission (Fig. 6a).

**Figure 7:**
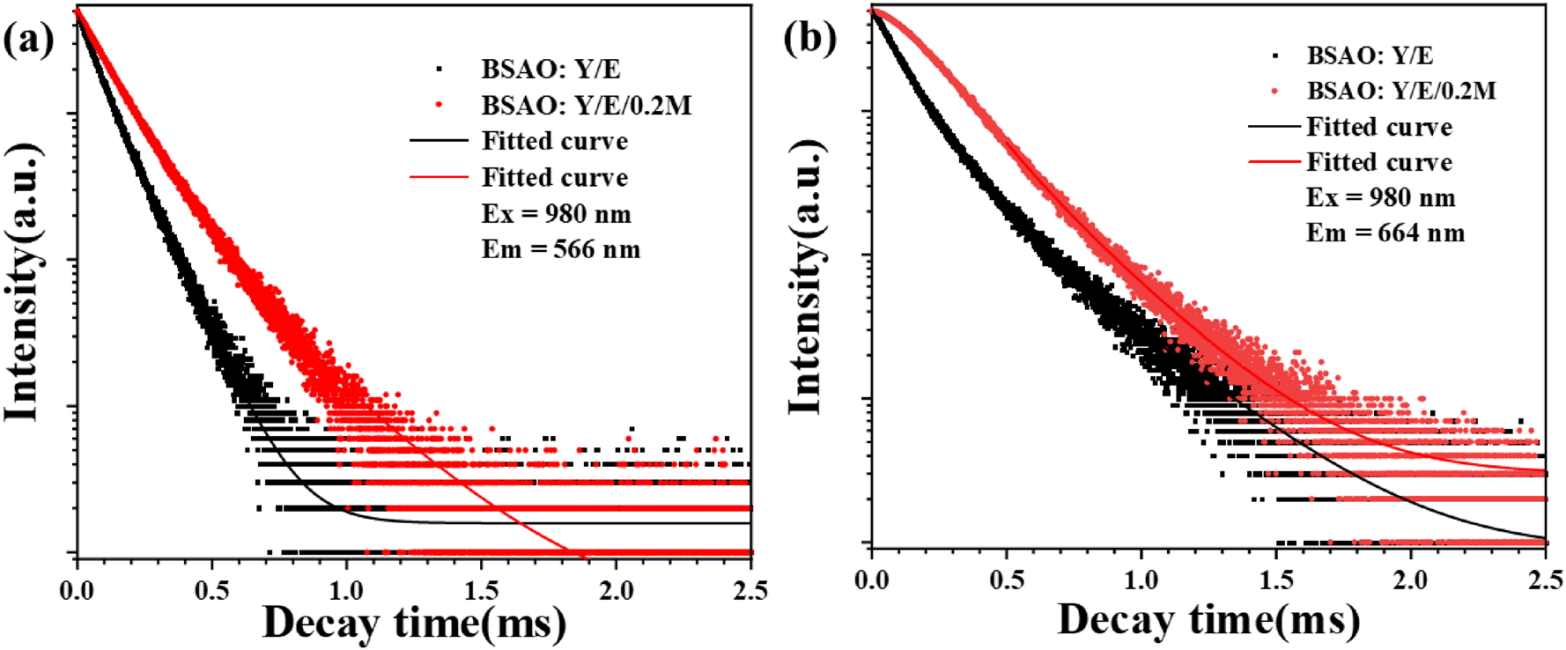
Fluorescence decay lifetime of BSAO: Y/E and BSAO: Y/E/0.2 M.

To further understand the effect of Mn^2+^ doping on the upconversion of BSAO: Y/E, Fig. 7a,b shows the upconversion green (566 nm) and red (664 nm) decay curves of BSAO: Y/E/*x*M (*x* = 0, 0.2) fluorescent material. BSAO: Y/E/*x*M (*x* = 0, 0.2) decay curves for both red (^4^F_9/2_ → ^4^I_15/2_) and green (^4^S_3/2_ → ^4^I_15/2_) light show biexponential decay curves, which are in good agreement with the second-order exponential decay mode. Since the curves exhibit double-exponential decay characteristics, the average fluorescence decay lifetime can be determined by the following equation^49^: $\tau =({A}_{1}{\tau }_{1}^{2}+{A}_{2}{\tau }_{2}^{2})/({A}_{1}{\tau }_{1}+{A}_{2}{\tau }_{2})$. *A*_1_ and *A*_2_ are constants, *τ* represents the decay time, and *τ*_1_ and *τ*_2_ represent the fast and slow exponential components. As can be seen from Fig. 7a,b, the fluorescence decay lifetime of 0.2Mn^2+^ ions doped with BSAO: Y/E is longer than that of BSAO: Y/E. The results show that the doping of Mn^2+^ increases the population number of Er^3+^ excited states. It is confirmed that the energy of the oxygen-vacancy induced defect band in the BSAO: Y/E/M phosphor is transferred to the excited state of the Er^3+^ ion^50,51^. Thus, the number of electrons in Er^3+^ ion transition from excited state to ground state is increased, and the UCL is enhanced.

**Figure 8:**
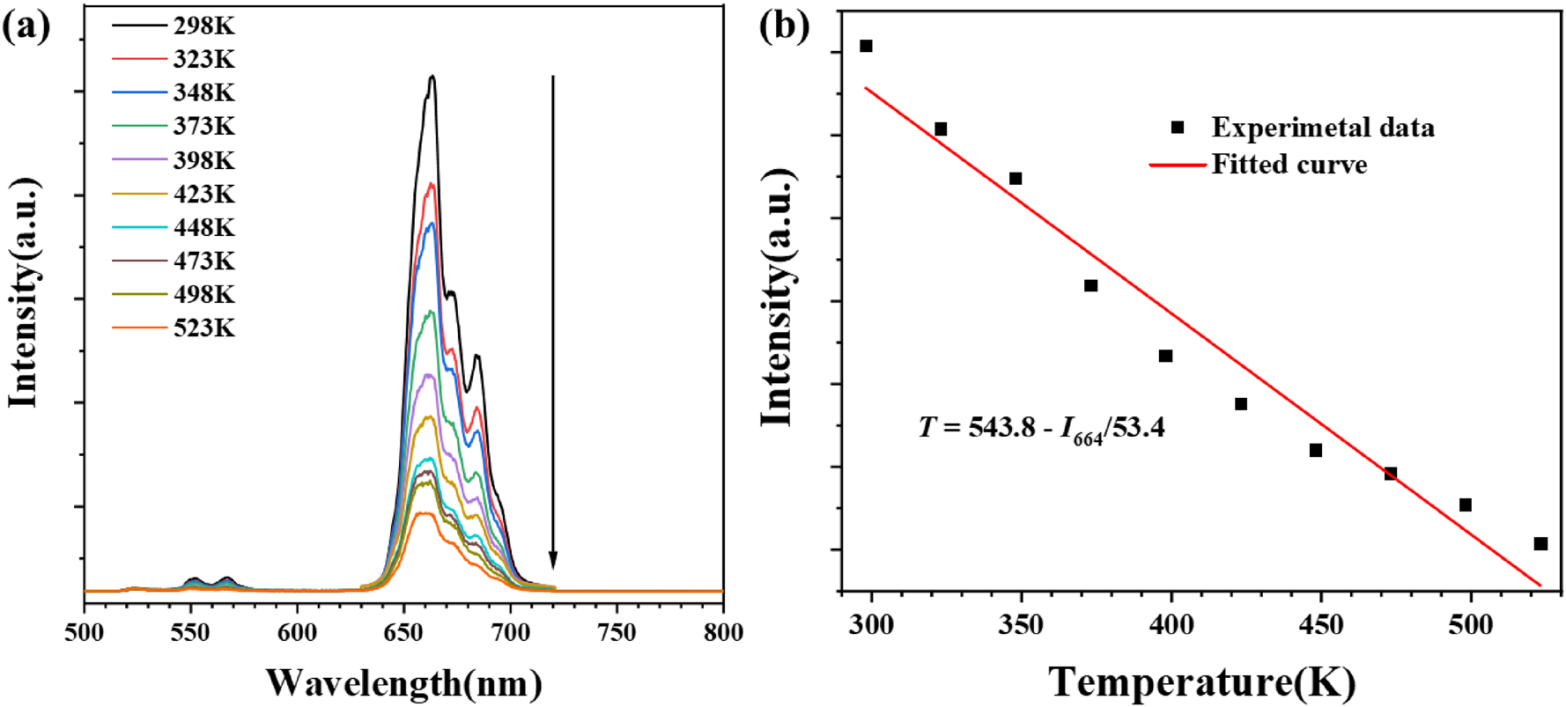
(**a**) The UCL spectra of BSAO: Y/E/0.2 M sample for different temperatures from 298 to 523 K. (**b**) The red integrated intensity as a function of the temperature.

BSAO: Y/E/0.2 M phosphor has a strong red UCL, making it an ideal candidate for single-parameter temperature sensors. In order to investigate the temperature sensitivity of BSAO: Y/E/0.2 M samples, the temperature dependence of the red UCL of the samples under 980 nm laser irradiation in the temperature range of 298–523 K is measured in Fig. 8a. The intensity of the red UCL decreases with increasing temperature. Figure 8b shows the temperature dependence of the red UCL integral intensity for BSAO: Y/E/0.2 M, where *T* and *I*_664_ are the absolute temperature and red integral intensity of the BSAO: Y/E/0.2 M samples, respectively, which can be fitted with *T* = 543.8−*I*_664_/53.4. The sensitivity (*S*) of the material is generally defined as *S* = (∂I_664_/∂T)/I_664_^52^. The maximum sensitivity of BSAO: Y/E/0.2 M phosphor as a temperature sensor at 523 K is 0.0247 K^−1^. The Mn^2+^ doped BSAO: Y/E red UCL phosphor has excellent potential for temperature sensing and can be used for practical non-contact sensing applications in harsh environments.

## Conclusion

This study successfully employed the solid-phase reaction strategy to synthesize a triple-doped BSAO: Y/E/M phosphor. Optimally doped with 0.20 Mn^2+^ ions, the sample showed a single upconversion red emission with a red-to-green integrated intensity ratio of 110.9, a 28.4-fold enhancement over the undoped BSAO: Y/E phosphor with Mn^2+^ ions. The intensity of red and green emissions is increased by 27.4 and 19.3 times, respectively. There are two pathways for red light enhancement. The first enhancement pathway: the energy of the ^4^F_7/2_ energy level of Er^3+^ is transferred to the defect band through energy transfer, and then the defect band is transferred to the ^2^H_11/2_, ^4^S_3/2_, and ^4^F_9/2_ energy levels through energy transfer to emit the corresponding green (530 and 566 nm) and red (664 nm) light. The second enhancement pathway: the non-radiative energy transfer of Er^3+^ to Mn^2+^ (^2^H _11/2_/^4^S_3/2_ → ^4^T_1_), followed by the back energy transfer to Er^3+^ (^4^T_1_ → ^4^F_9/2_). The maximum sensitivity of BSAO: Y/E/0.2 M phosphor as a temperature sensor at 523 K is 0.0247 K^−1^. The BSAO: Y/E/M phosphor has a wide range of applications in optical thermometry.

## Data Availability

All data generated or analysed during this study are included in this published article.
